# Reduced Fragmentation of IGFBP-2 and IGFBP-3 as a Potential Mechanism for Decreased Ratio of IGF-II to IGFBPs in Cerebrospinal Fluid in Response to Repeated Intrathecal Administration of Triamcinolone Acetonide in Patients With Multiple Sclerosis

**DOI:** 10.3389/fendo.2020.565557

**Published:** 2021-01-05

**Authors:** Andreas Hoeflich, Brit Fitzner, Christina Walz, Michael Hecker, Armin Tuchscherer, Julia Brenmoehl, Uwe Klaus Zettl

**Affiliations:** ^1^ Institute of Genome Biology, Leibniz Institute for Farm Animal Biology (FBN), Dummerstorf, Germany; ^2^ Department of Neurology, Neuroimmunological Section, University Medicine Rostock, Rostock, Germany; ^3^ Institute of Genetics and Biometry, Leibniz Institute for Farm Animal Biology (FBN), Dummerstorf, Germany

**Keywords:** insulin-like growth factor (IGF), insulin-like growth factor (IGF)-binding proteins (IGFBPs), cerebrospinal fluid (CSF), multiple sclerosis, IGFBP-fragment, triamcinolone acetonide

## Abstract

Multiple sclerosis (MS) is a chronic autoimmune disease of the brain and spinal cord causing a wide range of symptoms such as impaired walking capability, spasticity, fatigue, and pain. The insulin-like growth factor (IGF) system has regulatory functions for the induction of inflammatory pathways in experimental encephalomyelitis. We have therefore assessed expression and regulation of the IGF system on the level of IGFs and IGFBPs in serum and cerebrospinal fluid (CSF) in the course of four repeated triamcinolone acetonide (TCA) administrations in two female and four male MS patients. Sample series of 20 treatment cycles were analyzed. IGF-I and IGF-II were quantified by ELISAs, and IGFBPs were analyzed by quantitative Western ligand (qWLB) and Western immunoblotting (WIB) in order to differentiate intact and fragmented IGFBPs. The ratios of fragmented to intact IGFBP-2 and -3 were calculated in serum and CSF. Finally, the ratios of IGF-I and IGF-II to the total IGF-binding activity, quantified by qWLB, were determined as an indicator of IGF-related bioactivity. After the fourth TCA administration, the average level of IGF-I was increased in serum (p < 0.001). The increase of IGF-I concentrations in serum resulted in an increased ratio of IGF-I to IGFBPs in the circulation. By contrast in CSF, fragmentation of IGFBP-2 and IGFBP-3 and the ratio of IGF-II to intact IGFBPs were decreased at the fourth TCA administration (p < 0.01). Furthermore, reduced fragmentation of IGFBP-3 in CSF was accompanied by increased concentrations of intact IGFBP-3 (p < 0.001). We conclude that reduced fragmentation of IGFBPs and concomitant reduction of IGF-II to IGFBP ratios indicate regulation of bioactivity of IGF-II in CSF during repeated intrathecal TCA administration in MS patients.

## Introduction

### Multiple Sclerosis

Multiple sclerosis (MS) is a chronic progressive disease of the central nervous system that often manifests in young adulthood and particularly in women. MS is a very heterogeneous disease, with complex pathophysiology reflected by three different patterns of active white matter lesions (1). In the course of the disease, MS patients develop a variety of neurological symptoms such as depression, fatigue, paresis, and spasticity, the latter leading to severe impairment of the patients’ abilities, pain, and contractures (2). Therapeutic options for MS-related spasticity include oral applications of gamma-aminobutyric acid agonists, centrally acting α2 adrenergic agonists, postsynaptic muscle relaxants, and hydrochloride salts (2, 3). Another option for MS patients with predominantly spinal cord symptoms such as spasticity is the intrathecal injection of sustained-release steroids such as triamcinolone acetonide (TCA) (4). Due to its invasive application form, repeated TCA administration is restricted to highly selected patients and applied only at experienced MS centers.

### Relevance of the Insulin-Like Growth Factor System for MS

The insulin-like growth factor (IGF) system is particularly relevant in MS (5). In mice, disruption of the *Igf1* gene resulted in smaller brains, smaller amounts of oligodendrocytes, and deficits of myelination (6), whereas IGF-I transgenic mice had larger brains and increased myelin content in the brain (7). Therefore, the administration of IGF-I has been considered as an option for the treatment of MS. However, neither in rodent models nor in human MS patients, a significant effect of IGF-I alone or in combination with IGFBP-3 on demyelination or clinical impairment could be observed so far (8–11). Targeted expression of IGF-I in mouse brains in order to increase local IGF-I concentrations (12) did also not protect from experimental autoimmune encephalomyelitis (EAE). A very recent publication has demonstrated that, in fact, IGFs exert proinflammatory responses in EAE. This effect was shown to be dependent on the presence of IGF-I receptor and mediated *via* the Akt signaling pathway (13). At the cellular level, IGF-I induced the differentiation of T helper 17 (Th17) cells and therefore impacted the Th17 and regulatory T cell (Treg) balance (13), which is required for host defense and adequate immune tolerance. Notably, the Treg-Th17 balance is altered under conditions of autoimmune diseases, including MS (14, 15). Accordingly, it is essential to improve our understanding of how therapeutic approaches for MS may modulate the IGF system. The local activity of IGFs is regulated by hormone concentrations, IGF-I receptor density, and high-affinity IGF-binding proteins (IGFBPs) (5). According to the current concept of the IGF system, a proteolytic system triggers the controlled release of IGFs from IGFBPs, thereby mediating local and acute IGF effects *via* IGF-I receptors (16).

In order to assess the regulation of the IGF system in MS patients, we have quantified IGF and IGFBP concentrations in serum and CSF in response to repeated intrathecal TCA administration. We have also asked if proteolysis of IGFBPs can be postulated in serum or CSF, which may have relevance for the ratio of IGF to intact IGFBPs in both compartments.

## Methods

### Patients

**Figure 1:**
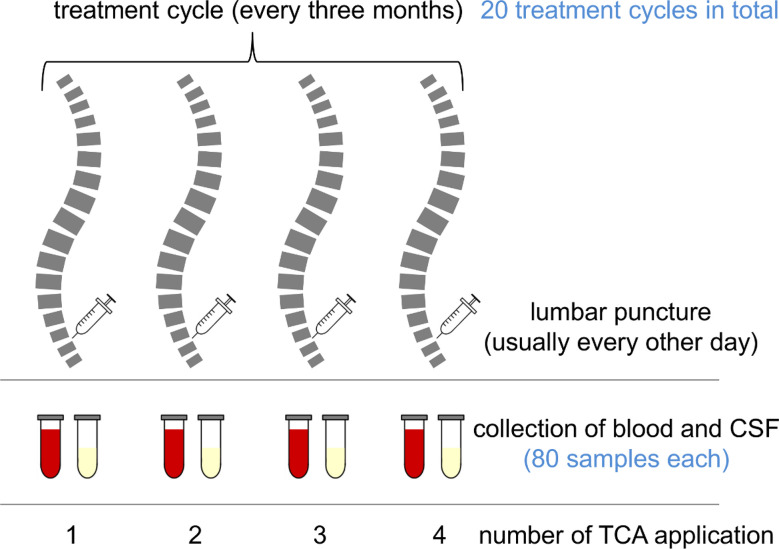
Chart of a treatment cycle and sample collection. Multiple sclerosis (MS) patients suffering from spasticity were treated with intrathecal triamcinolone acetonide (TCA) every 3 months. Each treatment cycle consisted of four TCA applications (usually every other day). The patients were lumbar-punctured, and to avoid CSF pressure differentials, the CSF was drained and replaced with the same amount of TCA solution. After the procedure, peripheral blood was drawn for routine laboratory analyses. Remaining sample material was stored in a biobank at −80°C.

In our biobank, we identified paired CSF and serum samples obtained as a by-product of the administration and monitoring of TCA therapy in MS patients with spasticity. The samples were collected in the years 2009–2012 and stored at −80°C until use. The patients gave their prior consent to the use of residual clinical samples for research purposes. The ethics committee of the University Medical Center Rostock approved the use of the samples for this study (approval A 2016-0088). We included only complete sample series representing the full treatment cycle (**Figure 1**) consisting of four applications of TCA (applied every second day in a dose range of 40–80 mg). CSF was collected immediately before intrathecal TCA injection and peripheral blood after lumbar puncture. Each patient received at least one treatment cycle of intrathecal TCA injections before the collection of the samples analyzed in this study. The previous treatment cycles of the patients ended about 3 months before. We examined paired CSF and serum sample series from 20 treatment cycles (six treatment cycles of two postmenopausal female patients and 14 treatment cycles of four male patients), resulting in the analysis of a total of 80 CSF and 80 serum samples. The patients were 52.9 ± 9.5 years old (mean ± standard deviation), had a median disease duration of 10 years (range: 4–26), and were characterized by relatively severe disability (Expanded Disability Status Scale score: 6.2 ± 1.1) and a body mass index of 28.3 ± 5.1. Only one of the patients received a disease-modifying treatment (glatiramer acetate) in addition to the intrathecal TCA injections.

### Analysis of Insulin-Like Growth Factors and Insulin-Like Growth Factor-Binding Proteins

IGF-I and IGF-II were studied in serum and CSF from all patients using commercial ELISAs according to the instructions of the manufacturer (product codes: E20 and E30, Mediagnost, Reutlingen, Germany). Concentrations of IGFBP-2 and -3 were determined in CSF and serum samples using quantitative Western ligand blotting, as described previously (17). Intraassay and interassay variations were smaller than 20% for the analytes, and limits of quantification were 0.25 ng for IGFBP-2 and 1 ng for IGFBP-3. Serial dilutions of human IGFBP-2 and -3 standards were used for the quantification of IGFBP-2 and IGFBP-3. In addition, Western immunoblotting was used for the study of the structural integrity of IGFBP-2 (32 kDa) and IGFBP-3 (band doublet between 40 and 43 kDa) in CSF and serum, also as described before (18). In brief, for the identification of IGFBP-2 and IGFBP-3 after transfer to polyvinylidene difluoride membranes, blots were incubated for 2 h at room temperature with specific antibodies (goat anti-human IGFBP-2, 1:1,000, Cat. No. SC-6002, Santa Cruz Biotechnology, Santa Cruz, CA, USA; rabbit anti-human IGFBP-3, 1:600, Cat. No. 13216, Cell Signaling Technology). After five consecutive washing steps for 5 min in Tris-buffered saline containing 0.05% Tween20, the membranes were incubated for 1 h with secondary antibodies (anti-goat or anti-rabbit IgG, 1:2,500). Again, recombinant human standards were used as positive controls, and molecular weight markers were used for estimating the molecular weights of IGFBP fragments. As a measure of IGFBP degradation, the ratio of fragmented to intact IGFBP was calculated for each lane. For the comparison of molar ratios, the concentrations of the IGF compounds were normalized by their molecular weight. Accordingly, IGF-I and -II levels were divided by 7.5, and IGFBP-2 and -3 levels were divided by 32 and 41.5, respectively.

### Statistical Analyses

**Figure 2:**
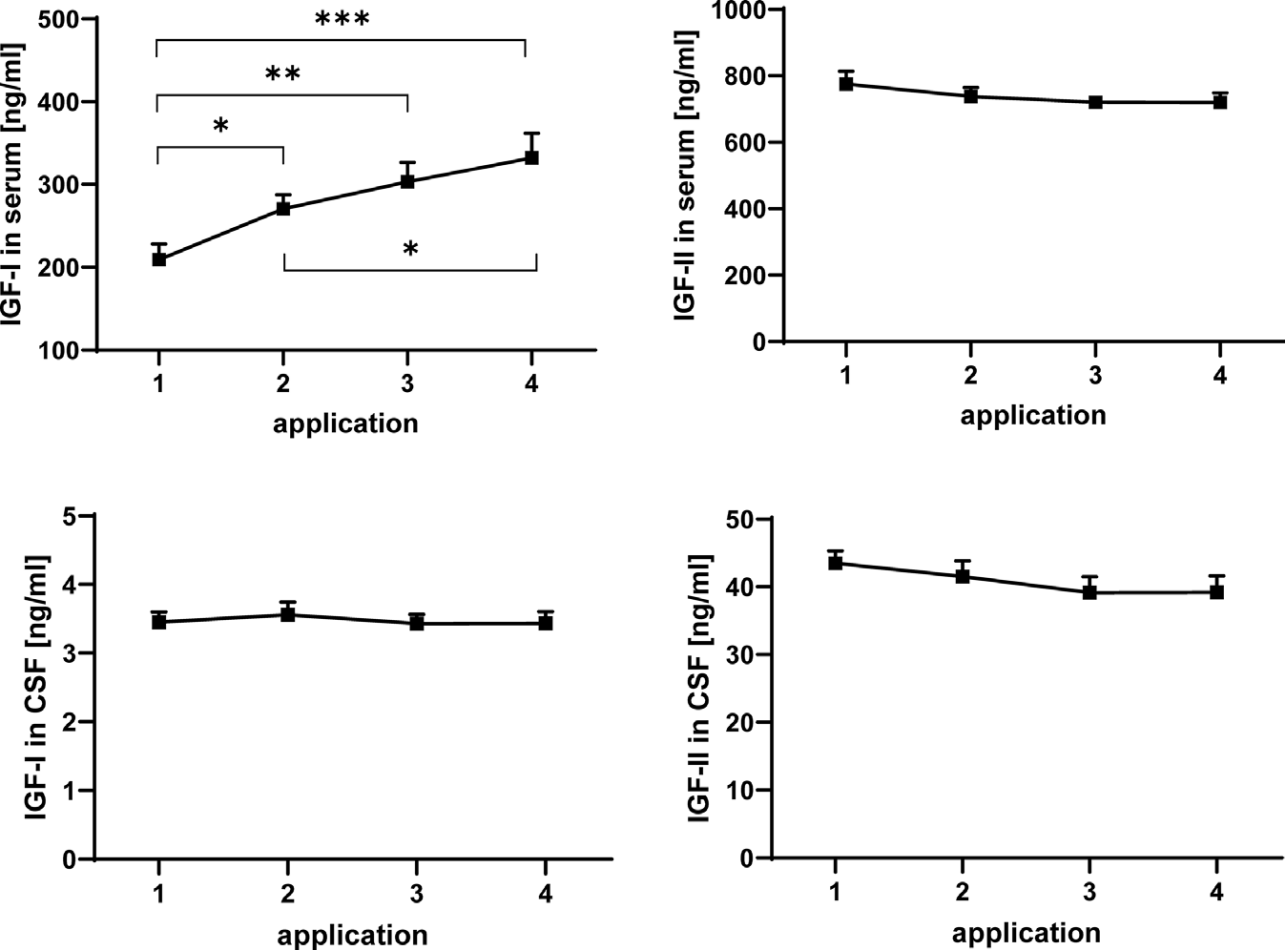
Concentrations of IGF-I (left panels) and IGF-II (right panels) in serum (upper panels) and CSF (lower panels) in MS patients at consecutive time points of intrathecal TCA injection. The data are based on sample series from 20 treatment cycles (14 cycles in four males and 6 cycles in two females) and are given as means ± SEM (significant differences are indicated; *p < 0.05; **p < 0.01; ***p < 0.001).

**Figure 3:**
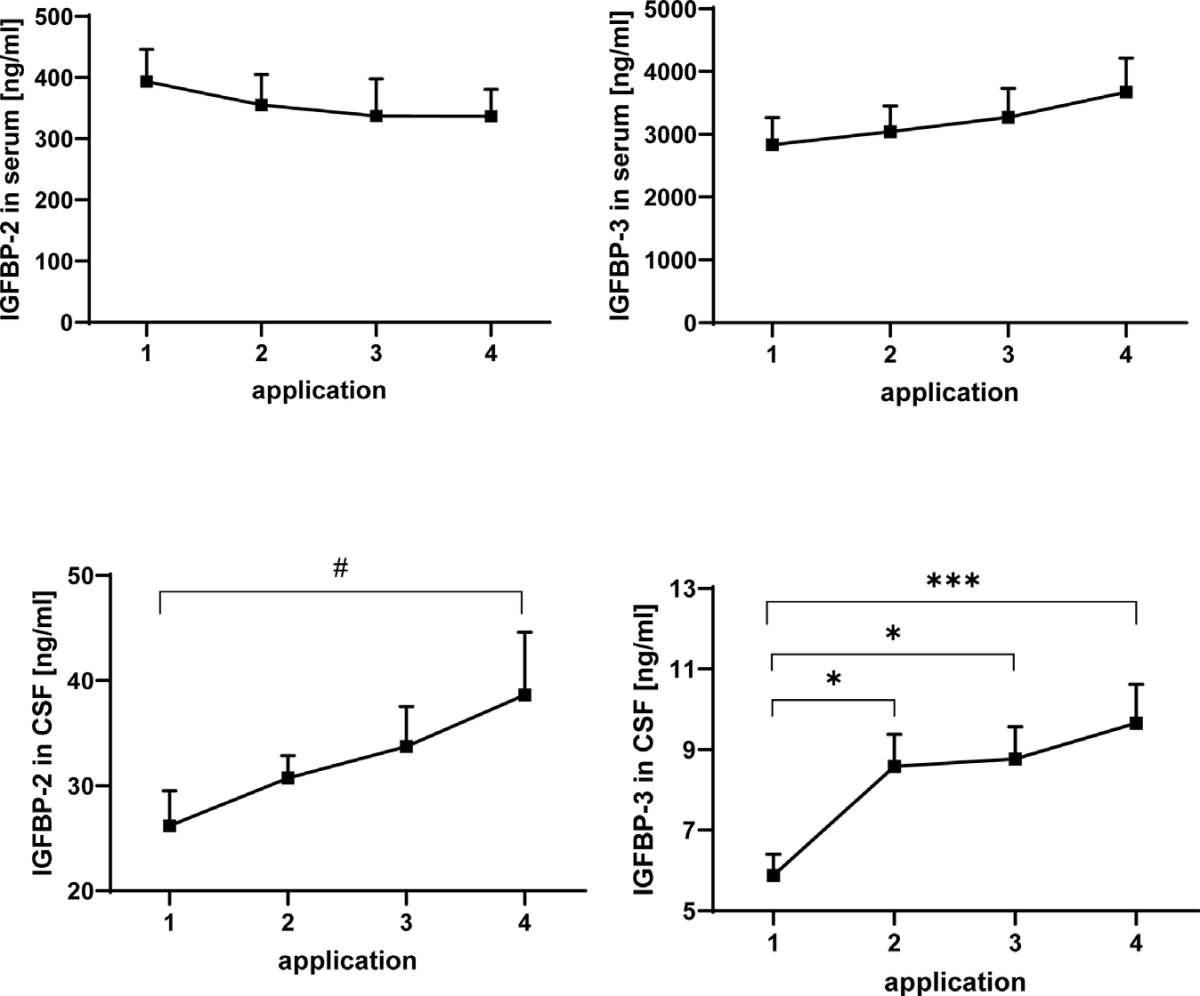
Concentrations of IGFBP-2 (left panels) and IGFBP-3 (right panels) in serum (upper panels) and CSF (lower panels) in MS patients at the time points of intrathecal TCA injection. The data are based on sample series from 20 treatment cycles (14 cycles in four males and 6 cycles in two females) and are given as means ± SEM (^#^p < 0.067; *p < 0.05; ***p < 0.001).

**Figure 4:**
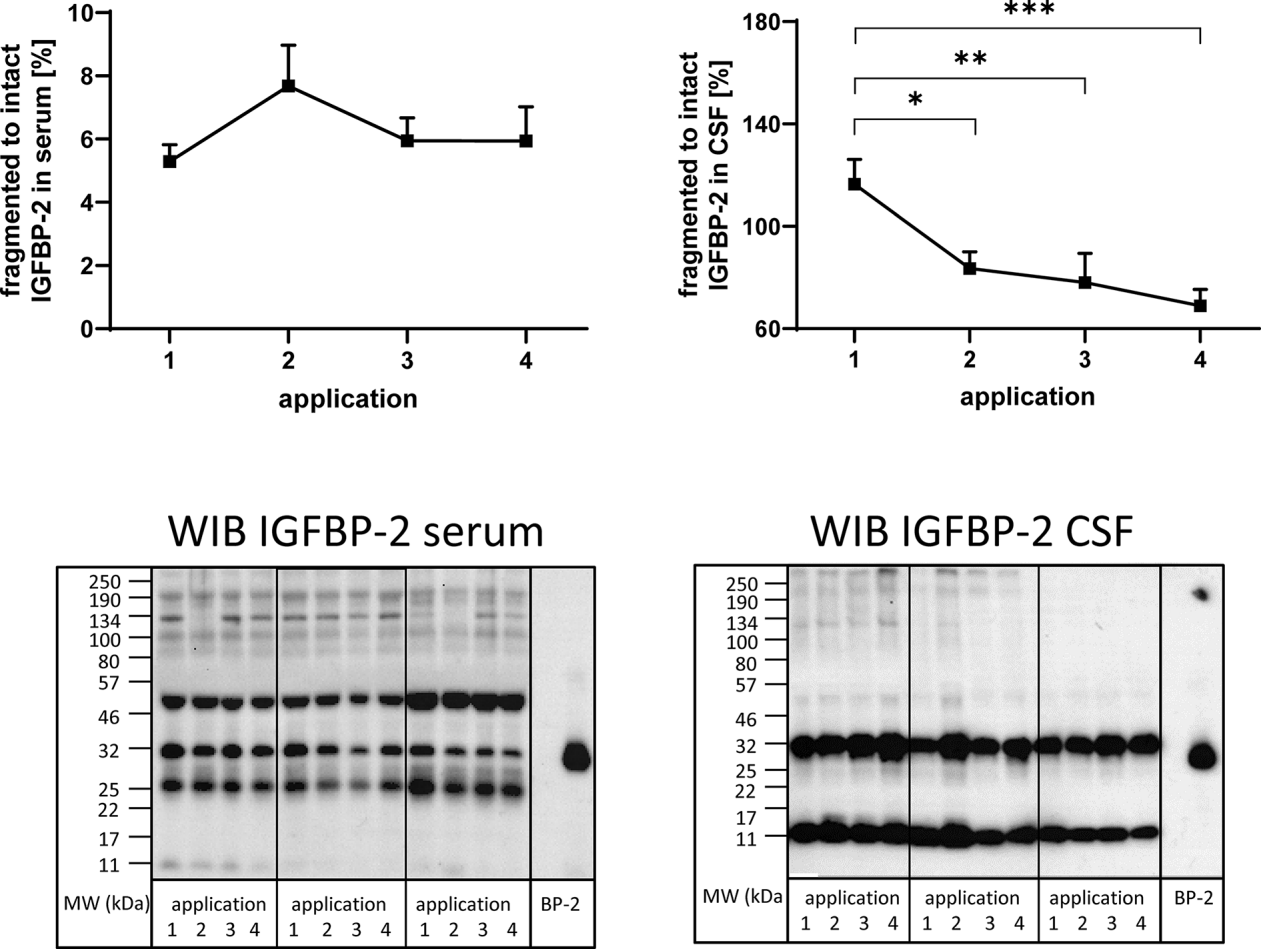
Fragmentation of IGFBP-2 in serum (left panel) and CSF (right panel) from MS patients in the course of repeated intrathecal TCA injections. A subset of samples from 14 treatment cycles (nine cycles in four males and five cycles in two females) was considered for this analysis. Fragmentation was calculated by the average ratio of fragmented (11 kDa) *versus* intact (32 kDa) IGFBP-2 and is presented in percentages (means ± SEM; significant differences are indicated; *p < 0.05; **p < 0.01; ***p < 0.001). On the lower panels examples for the Western immuno blot (WIB) are provided for the analysis of serum and CSF. All samples from a treatment cycles I to IV were loaded on the same gel and recombinant human IGFBP-2 (BP-2) was loaded as the positive control.

**Figure 5:**
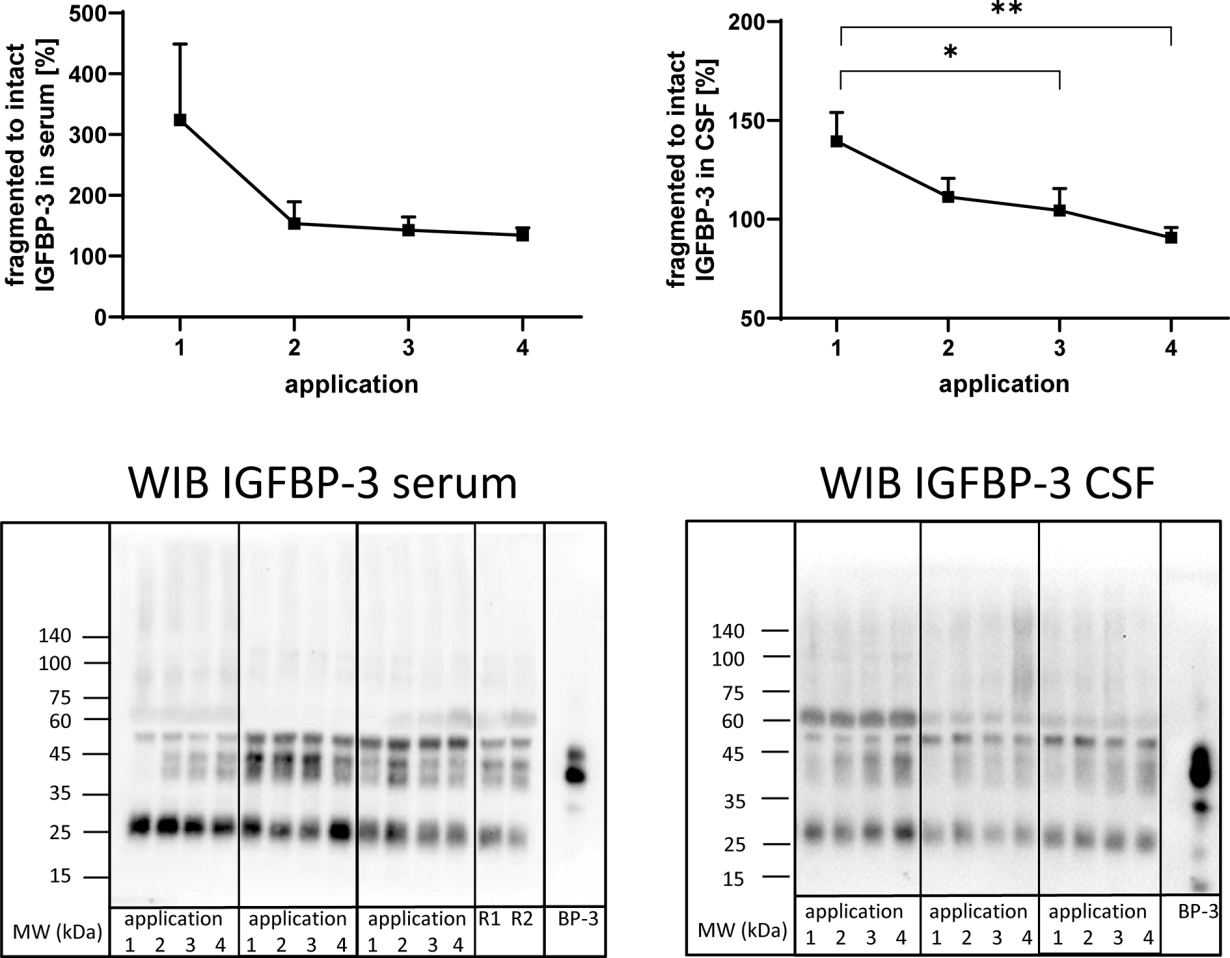
Fragmentation of IGFBP-3 in serum (left panel) and CSF (right panel) from MS patients at the time points of intrathecal TCA application. A subset of samples from 11 treatment cycles (seven cycles in four males and four cycles in two females) was used in this analysis. Fragmentation was calculated by the average ratio of fragmented (25 kDa) *versus* intact (40–43 kDa) IGFBP-3 and is presented in percentages (means ± SEM; *p < 0.05; **p < 0.01). On the lower panels examples for the Western immuno blot (WIB) are provided for the analysis of serum and CSF. All samples from treatment cycles I to IV were loaded on the same gel and recombinant human IGFBP-3 (BP-3) was loaded as the positive control. R1 and R2 represent technical replicate samples for normalization of different experiments.

**Figure 6:**
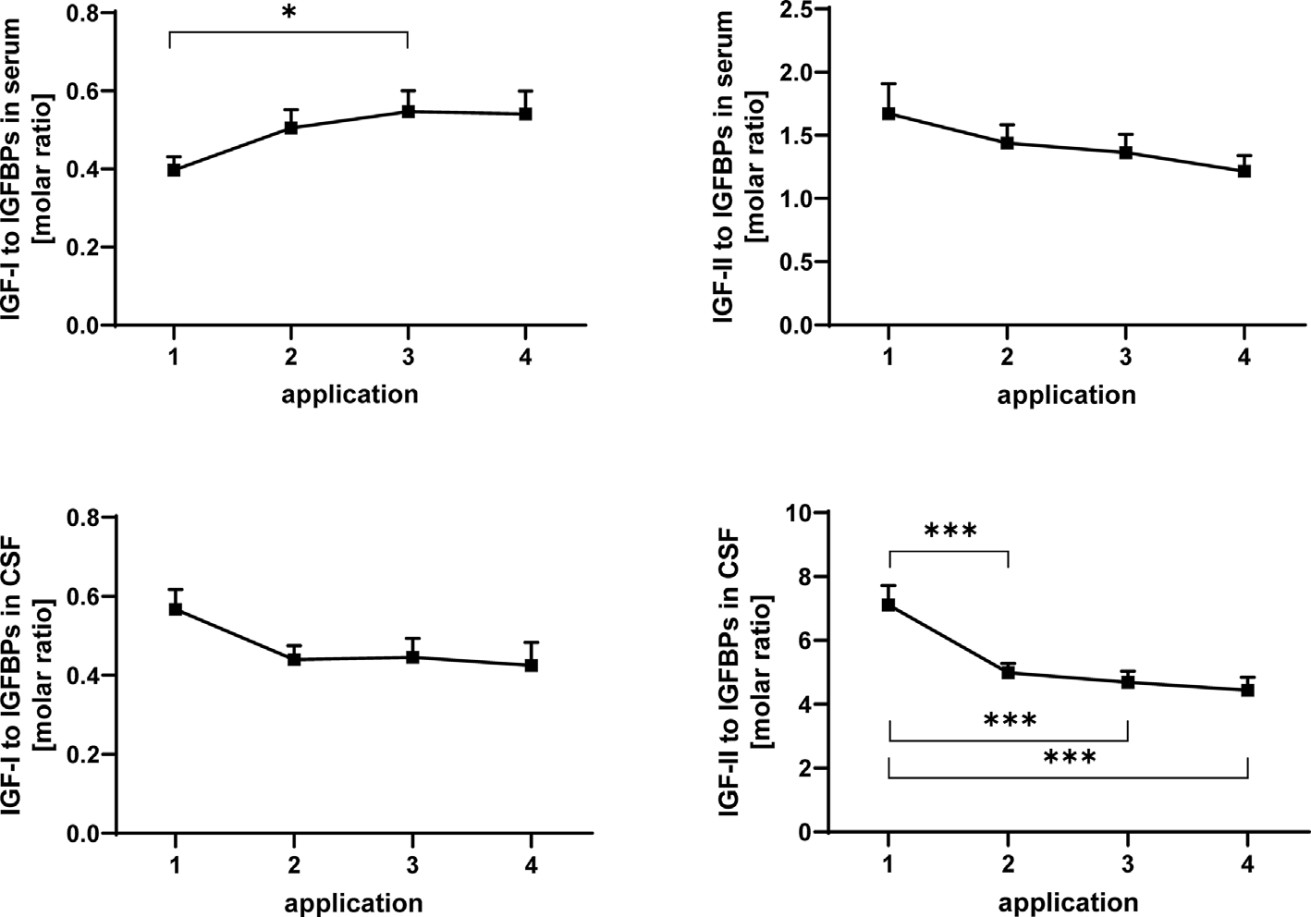
Molar ratios of IGF-I (left panels) and IGF-II (right panels) in serum (upper panels) and CSF (lower panels) to total IGF-binding activity detected by Western ligand blotting. The samples were obtained from MS patients with spasticity receiving treatment cycles of four intrathecal TCA applications. Total IGFBP activity is composed of IGFBP-2 and IGFBP-3. The plot depicts the average change in molar ratios over 20 treatment cycles (14 cycles in four males and 6 cycles in two females). Data are given as means ± SEM (*p < 0.05; ***p < 0.001).

The data analysis was performed using SAS software, Version 9.4 for Windows (SAS Institute Inc., Cary, NC, USA). Descriptive statistics and tests for normality were calculated with the UNIVARIATE procedure of Base SAS software. CSF and serum data were approximately normally distributed and were analyzed by repeated measurement analyses of variance using the MIXED procedure of SAS/STAT software. The repeated measurement ANOVA model for CSF and serum data (statistical model #1) contained the fixed factor application (levels: I, II, III, and IV) and the covariate treatment cycle. Repeated measures on the same patient (treatment cycle, application) were taken into account by the REPEATED statement of the MIXED procedure using the SUBJECT=patient option to define the blocks of the block-diagonal residual covariance matrix and the TYPE=CS option to define their covariance structure. Least-squares means (LS-means) and their standard errors (SE) were computed for each fixed effect in the model, and all pairwise differences of these LS-means were tested by the Tukey-Kramer test, a procedure for pairwise multiple comparisons. The SLICE statement of the MIXED procedure was used for performing partitioned analyses of the LS-means. With respect to the small number of patients included in the present study and the imbalanced gender contribution, gender was not included in the statistical analysis presented in **Figures 2**–**6**. Nevertheless, the effect of gender was considered by extending the above statistical model #1 by *gender* as a fixed factor in a separate statistical analysis (statistical model #2). Test results were considered significant if p < 0.05.

## Results

### Analysis of Insulin-Like Growth Factor-I and Insulin-Like Growth Factor-II in Serum and Cerebrospinal Fluid

The concentrations of IGF-I and -II in serum and CSF were quantified by ELISA. Repeated TCA application had a significant enhancing effect on the concentration of IGF-I in serum (**Figure 2**) when all samples were considered in the analysis (application 1 *versus* application 4: p < 0.001). Within the treatment cycles a progressive increase of serum IGF-I can be described, with significant increases between application #1 and #2 (p < 0.05) and additional increases between application #2 and #4 (p < 0.05). If gender was included in the statistical model (model #2), the male groups was characterized by higher concentrations of IGF-I in all serum samples (p < 0.01; data not shown). In CSF from the same patients, application of TCA had no effect on the IGF-I. In addition, significant effects of repeated TCA injections could not be observed on the concentrations of IGF-II in serum or CSF.

### Analysis of Insulin-Like Growth Factor-Binding Proteins in Serum and Cerebrospinal Fluid by Western Ligand Blotting

IGFBPs were analyzed by Western ligand blotting in serum and CSF of TCA-treated MS patients (**Figure 3**). Similar to IGF-I, IGFBP-3 concentrations in serum were higher in males than in females (statistical model #2: p < 0.05, data not shown). While in serum the concentrations of IGFBP-2 and IGFBP-3 were not altered in response to intrathecal TCA injections, the average IGFBP-3 levels in CSF were elevated from TCA application #1 to TCA application #4 (p < 0.001). The increase of IGFBP concentrations in the CSF was evident in the sample series of both genders, even though statistical significance was reached only for IGFBP-3 within the female group (p < 0.05). A significant increase of IGFBP-3 in CSF was present already at the time of application #2 if compared to application #1 but not further increased to a significant extent between application #2 and #4. The increase of IGFBP-2 in response to TCA application between application #1 and #4 only reached border significance (p < 0.067) but was identified if the statistical model included gender as a fixed effect (statistical model #2: application #1 *versus* application #4: p < 0.05).

### Identification of Insulin-Like Growth Factor-Binding Protein Fragments and Ratio of Fragmented Versus Intact Insulin-Like Growth Factor-Binding Proteins

In order to investigate the presence of IGFBP fragments in serum and CSF, Western immunoblotting was used. IGFBP-2-related immunoreactivity was present at four and two different molecular weights in serum and CSF (**Figure 4**), respectively. In serum, bands around 50, 32, 25, and 11 kDa were identified, while in CSF, bands around 32 and 11 kDa were detected. The ratio of fragmented (11 kDa) to intact (32 kDa) IGFBP-2 was higher in CSF than in serum by more than one order of magnitude (p < 0.001). As an effect of repeated TCA administration, the ratio of fragmented (11 kDa) to intact (32 kDa) IGFBP-2 decreased in CSF (application 1 *versus* 2, 3, or 4: p < 0.01). The reduction of IGFBP-2 fragmentation in CSF in the course of the TCA treatment cycle was also significant when the female group was assessed separately (p < 0.01; data not shown). The ratio of the 25 kDa or 50 kDa IGFBP-2 band to intact IGFBP-2 was not affected by TCA application (data not shown).

Antibodies directed against IGFBP-3 detected intact IGFBP-3 (40-43 kDa) and additional bands characterized by higher (≈55 kDa) or lower (≈27 kDa) molecular weight in serum and CSF (**Figure 5**). As an effect of repeated intrathecal TCA application, the ratios of fragmented to intact IGFBP-3 decreased in CSF samples from TCA applications #3 and #4 compared to application #1 (p < 0.05).

### The Molar Ratios of Insulin-Like Growth Factor-I and Insulin-Like Growth Factor-II to the Sum of Insulin-Like Growth Factor-Binding Proteins Quantified by Western Ligand Blot

In serum, the ratio of IGF-I to IGFBPs was significantly (p < 0.05) increased between application #1 and #3 (**Figure 6**). The ratio of IGF-II to the sum of IGFBP-2 and -3 (**Figure 6**) was significantly reduced in CSF after the initial TCA application (p < 0.01). This reduction (TCA application #1 compared to applications #3 and #4) was also significant when the samples from men and women were analyzed separately (p < 0.05; data not shown).

## Discussion

IGF-I is required for brain growth and development, and the lack of IGF-I in knockout mice resulted in reduced brain growth and hypomyelination (6). In addition, IGF-I, as an effector of Th17/Treg cell balance, induced proinflammatory responses in EAE (13). Therefore, IGF-I is not only considered as a neuroprotective agent but is also discussed concerning health-related fitness in MS patients after aerobic training (19). Accordingly, substantial reasons argue for the assessment of the IGF system in MS patients, both in CSF and in the circulation. Here, we analyzed the dynamics of IGF-I and -II and IGFBPs in serum and CSF in male and female MS patients in response to four consecutive intrathecal TCA injections. In order to estimate IGF-related bioactivity in serum and CSF, we calculated the molar ratios of IGF to the sum of intact IGFBPs detected by quantitative Western ligand blot in both compartments.

### Compounds From the Insulin-Like Growth Factor System in Serum and Cerebrospinal Fluid From Multiple Sclerosi Patients

Our results on the concentrations of IGFs and IGFBPs in serum generally correspond to previous findings in MS patients (20, 21). Compared to other studies in MS patients (22–24), but also to published reference concentrations (25), we found higher IGF-I concentrations in the serum of our patients. The reason for this could be related to differences in severity and stage of the disease, therapeutic interventions, different age, and gender distributions, and, last but not least, to different analytical techniques. As we have identified an inducing effect of intrathecal TCA administration on serum concentrations of IGF-I, it is possible that repeated TCA injections may have long-lasting enhancing effects on serum IGF-I concentrations in MS patients.

For CSF, concentrations of IGFBP-3 have already been published in a similar range as we found in this study (20). By contrast, IGFBP-2 concentrations in the CSF are 5- to 10-fold lower in the present study compared to other studies in MS patients (20, 26). In these studies, IGFBP-2 ELISAs were used. Therefore, the strong differences could be related to the different methods used and/or to the presence of IGFBP-2 fragments in the CSF (27), which potentially were included in the measurement by quantitative ELISA but not in quantitative Western ligand blotting. Between 3 and 40 years of age, the CSF concentrations of IGFBP-3 range between 14 and 22 ng/ml (28). However, CSF IGFBP-3 levels decrease with age (28), possibly explaining why lower concentrations (5–13 ng/ml) were observed in the patients, which were up to 67 years old. The measured concentrations of CSF IGF-I (3.4–4 ng/ml) and -II (37–49 ng/ml) were higher (20) or in a similar range (23, 27) compared to published results from the literature.

### Association of Age and Gender With the Insulin-Like Growth Factor System

In this study, four male and two female patients aged between 40 and 67 years were included. Due to the small number of patients, gender-specific differences could not be clearly delineated. However, according to published reference levels (25), males (at the age of 51–55 years: 119 ng/ml) have slightly higher concentrations of IGF-I in serum than females (at the age of 51–55 years: 110 ng/ml). Moreover, as an effect of age, the level of IGF-I in serum is roughly 10 ng/ml lower per decade of life (29). This may be related to growth hormone (GH) secretion, which is altered during chronic or acute illness (30) and is known to control both IGF-I and IGFBP-3 levels. Interestingly, in glial cells, sex steroids and glucocorticoids have been shown to differentially regulate expression of the IGF system (31). Unfortunately, IGF-II mRNA expression was not assessed in the cited study (31). Since TCA is detectable in the periphery after intrathecal administration (32), differential steroid effects on IGF secretion can be assumed in the periphery as well. Effects of steroids on the expression and secretion of IGF-I have been already demonstrated in animal models (33) and healthy subjects (34).

### Effects of Triamcinolone Acetonide Application

Serum IGF-I concentrations increased in response to repeated TCA applications. This effect was also significant in the male subgroup. A previous study showed that after aerobic training, the serum concentrations of IGF-I in MS patients are significantly correlated with muscle strength (left and right-hand strength, quadriceps strength) and walking speed (19). Low levels of IGF-I in the circulation were further associated with fatigue and cognitive impairment in MS patients (24). The positive effects of repeated TCA injections on serum concentrations of IGF-I and on the ratio of IGF-I to IGFBPs in serum from MS patients may thus have relevance for physical and cognitive functions. A potential relation to the finding that repeated application of TCA significantly increased walking distance in MS patients (35) may be addressed in future multicentric studies comprising larger patient cohorts.

In CSF, but not in the circulation, repeated intrathecal application of TCA evoked increased concentrations of IGFBP-3 and with borderline significance also of IGFBP-2. Degradation products were seen for both IGFBP-2 and -3. The elevated intrathecal levels of IGFBP-2 and -3 in the course of TCA treatment were reflected by reduced levels of distinct IGFBP-2 and -3 fragments. Proteolytic degradation of IGFBPs in the periphery is part of the physiological growth control in human development (36–38) as well as in cancer (39).

For IGFBP-2, proteolytic fragments characterized by molecular weights of 12 and 23 kDa have been described (39). Notably, both fragments can be formed by the activity of PAPP-A (40). By contrast, the nature of IGFBP-2 related immunoreactivity around 50 kDa is less clear and further investigation is required to clarify the identity of this band, although no biomarker content appeared to be connected with this signal in this study. For IGFBP-3, which is cleaved by PAPP-A2 (16), a proteolytic fragment with molecular weight smaller than 30 kDa and bigger than 25 kDa also was describe before (18, 41). Since the concentrations of IGFBP-2 and -3 in serum were in a normal range as discussed above, we have no direct evidence to assume that the substantial amounts IGFBP-fragments are due to unspecific degradation during long-term storage in this compartment. Instead, endogenous proteases may be responsible for a high turnover of intact IGFBPs as part of physiological and conditional control of IGF-system in the circulation (16). In a recent study, PAPP-A was identified also in CSF from diabetic patients with and without diabetic polyneuropathy and in control participants (42). The study by Kallestrup et al. (42) revealed a positive correlation between the Neuropathy Rank Sum Score (NRSS) and proteolytic activity (i.e., concentration of fragmented IGFBP) in CSF. Since IGFBP proteolysis in diabetic patients is positively correlated with NRSS, reduced IGFBP proteolysis may contribute to beneficial effects of repeated TCA administration in MS patients. Subsequent studies are warranted for confirmation and closer inspection of the underlying molecular events.

In order to model the interactions between IGFs and IGFBPs, we calculated the ratios of both growth factors to the binding activity detected by Western ligand blotting. As an effect of TCA application, the ratio of IGF-II to the IGF-binding activity (intact IGFBP-2 plus intact IGFBP-3) was reduced at application #4 compared to application #1. Since the concentrations of IGF-II in CSF did not change significantly during TCA treatment, we can assume that the ratio of IGF-II to IGF-binding activity is regulated in response to intrathecal TCA administration at the level of IGFBPs and more specifically at the level of IGFBP proteolysis. From the reduced ratio of IGF-II to IGF-binding activity in CSF, it might be appropriate to assume lower IGF-bioactivity at TCA application #4. With respect to the important and novel findings by DiToro et al. (13), it is possible to conclude here that reduction of IGF-related bioactivity could block inflammatory pathways also in MS patients. In fact, a direct relation between proteolytic activity and bioactivity of IGF-I has been established in pleural fluid (18). Although in CSF from diabetic patients, elevated bioactivity of IGF-I in the presence of elevated IGFBP proteolysis could not be confirmed (42).

Our retrospective study has several limitations. Lumbar puncture is an invasive procedure that is rarely performed, e.g. to confirm the diagnosis of MS. An exception is the intrathecal administration of TCA, which can be considered in a small number of MS patients with spasticity for whom no other therapies are effective. Therefore, the number of patients that could be included in this study was limited and imbalanced in terms of sex and age distribution. Furthermore, healthy controls could not be included, and thus, it is challenging to interpret absolute hormone levels determined in MS patients. Therefore, quantitative data were discussed in comparison to published hormone concentrations in serum and CSF. Moreover, we only addressed IGFBPs detected by Western ligand blotting but did not include low-abundant IGFBPs because serum and CSF were not available in quantities sufficient for multiple ELISAs. Proteolysis of IGFBPs in serum and even more in CSF has received not much attention and we do not have specific knowledge about preclinical conditions affecting the concentrations of intact *versus* fragmented IGFBPs in either matrix, be it fresh or long-term stored. It is also unknown if the substantial reductions of IGF-II to IGFBP ratio in CSF are biologically relevant since IGF-II is in high molar excess to the IGFBPs in CSF. It would thus be necessary to study IGF-II related biological activity in CSF in future studies. Finally, future studies will also have to assess the roles of PAPP-A and PAPP-A2 for the control of IGF-related bioactivity in order to test hypotheses developed or supported by the present study.

To summarize, in response to repeated intrathecal TCA administration, the IGF system is regulated differently in serum and CSF: IGF-I concentration and the ratio of IGF-I to intact IGFBPs is elevated in serum, while IGFBP-3 and, at least in tendency, IGFBP-2 levels increase in response to TCA administration in CSF. The increase of intact IGFBP-3 concentrations is reflected by reduced levels of respective IGFBP fragments in CSF and by reductions of the IGF-II to IGF-binding activity ratios during the treatment cycles. We hypothesize that intrathecally administered TCA influences IGF-II-related bioactivity either by controlling IGFBP fragmentation in CSF and/or by controlling the molar ratio of IGF-II to IGFBPs. Accordingly intrathecal injection of TCA differentially regulates the systemic and central IGF-system in MS patients.

## Data Availability Statement

The original contributions presented in the study are included in the article/supplementary materials; further inquiries can be directed to the corresponding authors.

## Ethics Statement

The studies involving human participants were reviewed and approved by the ethics committee of the University Medical Center Rostock and approved the use of the samples for this study (Approval A 2016-0088). Written informed consent for participation was not required for this study in accordance with the national legislation and the institutional requirements.

## Author Contributions

CW generated the data. AH, AT, and BF produced the figures. AT performed the statistical analysis. All authors contributed to the article and approved the submitted version.

## Funding

The publication of this article was funded by the Open Access Fund of the Leibniz Association and the Open Access Fund of the Leibniz Institute for Farm Animal Biology (FBN).

## Conflict of Interest

AH is related to Ligandis UG. MH received speaking fees and travel funds from Bayer HealthCare, Biogen, Merck, Novartis, and Teva. UZ received research support as well as speaking fees and travel funds from Almirall, Bayer HealthCare, Biogen, Merck Serono, Novartis, Sanofi Genzyme, and Teva. UZ received financial support for other research activities from Almirall, Bayer HealthCare, Biogen, Merck Serono, Novartis, Sanofi Genzyme, and Teva.

However, these funders had no role in the study design, data collection and analysis, decision to publish, or preparation of the present manuscript.

The remaining authors declare that the research was conducted in the absence of any commercial or financial relationships that could be construed as a potential conflict of interest.
